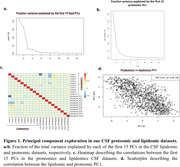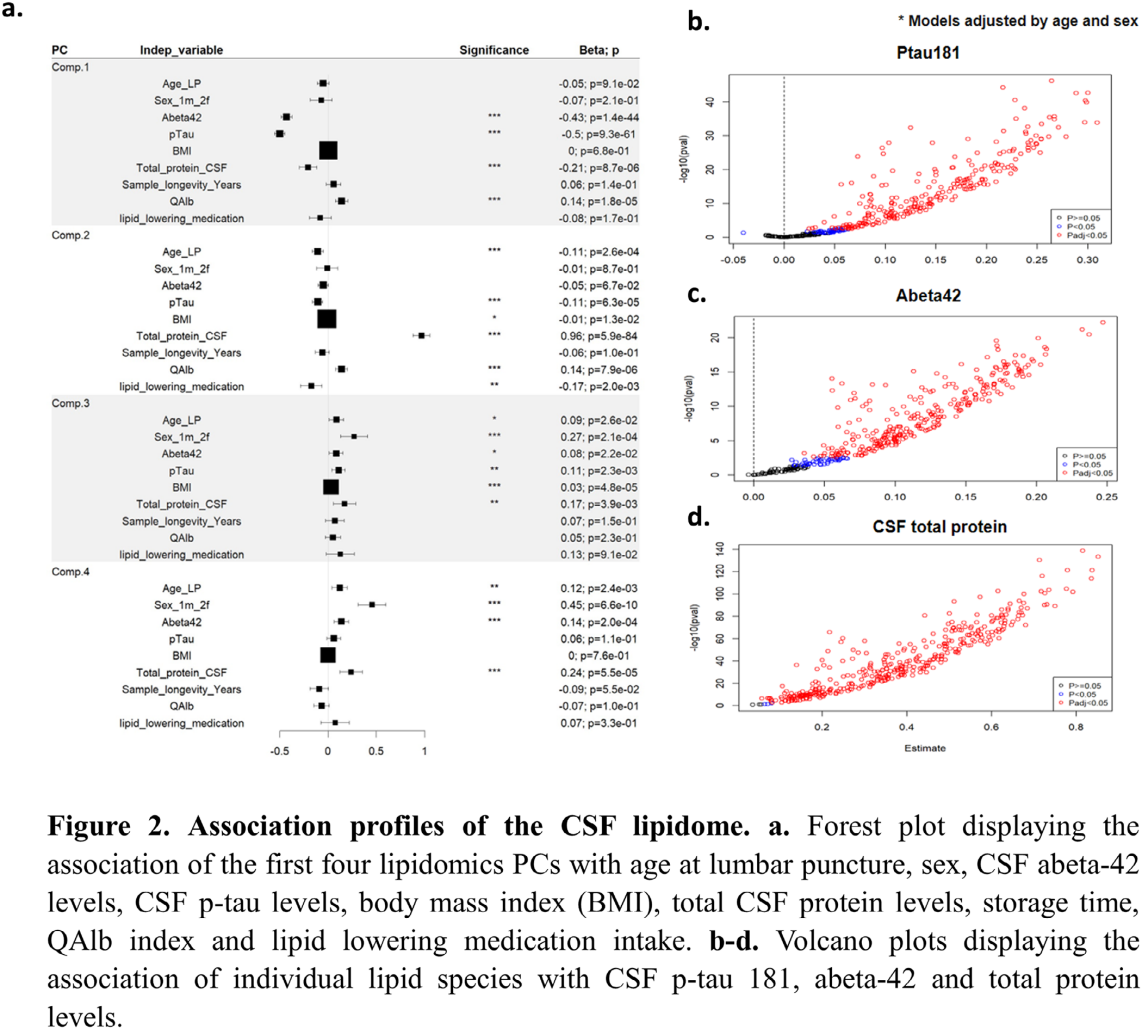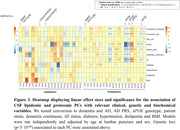# Deciphering Alzheimer's Disease Complexity: Integrative Analysis of CSF Proteomic and Lipidomic Data through Dimensionality Reduction

**DOI:** 10.1002/alz.088693

**Published:** 2025-01-09

**Authors:** Pablo García‐González, Raquel Puerta, Jonas Dehairs, Itziar de Rojas, Laura Montrreal, Vanesa Verónica Pytel, Marta Marquié, Maitee Rosende‐Roca, Asif Emon, Bart Smets, Adelina Orellana, Lluís Tárraga, Mercè Boada, Johannes V Swinnen, Victoria Fernández, Alfredo Cabrera Socorro, Agustin Ruiz

**Affiliations:** ^1^ Ace Alzheimer Center Barcelona – International University of Catalunya (UIC), Barcelona Spain; ^2^ Laboratory of Lipid Metabolism and Cancer, Department of Oncology, KU Leuven, Lueven Belgium; ^3^ CIBERNED, Network Center for Biomedical Research in Neurodegenerative Diseases, National Institute of Health Carlos III, Madrid Spain; ^4^ Janssen Research & Development, A Division of Janssen Pharmaceutica, Neuroscience Therapeutic Area, Beerse Belgium; ^5^ Glenn Biggs Institute for Alzheimer's & Neurodegenerative Diseases, University of Texas Health Science Center at San Antonio, San Antonio, TX USA

## Abstract

**Background:**

Alzheimer’s Disease (AD) is a complex disorder and much of its etiopathology is still unknown. Here, we applied dimensionality reduction methods to disentangle cyptic patterns in CSF proteomic and lipidomic data.

**Method:**

We studied 1121 CSF samples using targeted lipidomics based on liquid chromatography (LC)‐MS/MS (mass spectrometry), generated by Lipometrix (Lueven, Belgium), and proteomic data generated by Somalogic (Boulder, Colorado) using the SOMAscan 7k Assay. We independently computed the principal components for the proteomic and lipidomic datasets using good quality lipids (N=388) and proteins (N=2469). CSF samples were obtained by lumbar punctures at ACE Alzheimer Center (Barcelona, Spain), including patients at different points of the dementia continuum (SCD, MCI and dementia). Principal components were calculated using the princomp() function in R.

**Result:**

PC1 explained a substantial fraction of the variance in lipidomic (∼40%) and proteomic (∼60%) datasets and was highly correlated between both omics (R2=0.43; p=2.3·10^‐138^, Figure 1). We explored the association profile of the PCs to AD risk factors (age, sex, APOE, PRS, diabetes, hypertension, dyslipidemia, BMI), endophenotypes (abeta, tau), disease progression and total protein in the CSF. PC1, as well as the individual lipid species, were strongly associated with abeta, tau and total CSF protein levels (Figure 2). Finally, we conducted a GWAS of the first 20 lipidomic and proteomic PCs. PC1 displayed a GWS signal at chr3q28 in both omics. This region has been previously linked with CSF tau levels and brain morphology. Subsequent lipidomic PCs were associated with variants in the FADS1/FADS2 locus, while other proteomic PCs were associated with variants in the APOE locus (Figure 3).

**Conclusion:**

Our findings revealed a shared major contributor to the variance in CSF between lipidomic and proteomic data, which is related to the tau, abeta and total protein signature, and identified a QTL associated to both the lipidomic and proteomic PC1. Accounting for this major variance contributor may enhance future studies involving CSF biomarkers. Subsequent principal components had specific association profiles to AD risk factors and endophenotypes.